# Heart-specific expression of the green fluorescent protein gene in avian embryos by administration of recombinant adenovirus type-5 vector into the embryonic blood vessel

**DOI:** 10.3389/fphys.2024.1467489

**Published:** 2024-09-27

**Authors:** Wonjun Choi, Joonbum Lee, Dong-Hwan Kim, Evan Ma, Yeunsu Suh, Sang-Suk Lee, Kichoon Lee

**Affiliations:** ^1^ Department of Animal Sciences, The Ohio State University, Columbus, OH, United States; ^2^ Department of Animal Science, College of Natural Resources & Life Science, Pusan National University, Miryang, Republic of Korea; ^3^ Department of Animal Science and Technology, Sunchon National University, Suncheon, Republic of Korea

**Keywords:** adenovirus type 5, green fluorescent protein expression, quail embryo, heart, chicken embryo

## Abstract

Genetic modification *in vivo* could provide direct functions of genes that could potentially contribute to diverse areas of research including genetics, developmental biology, and physiology. It has been reported that genes of interest could be introduced via recombinant adenovirus type 5 (Ad5) in poultry. Successful gene delivery to mammal fetuses *in utero* promises substantial progress in clinical and developmental biology, but it is limited because of difficulties in injecting specific sites and invasiveness. On the other hand, developing avian embryos are easily accessible by making a window on the eggshell. Therefore, the objective of this study is to determine permissive embryonic stages for gene transfer into specific avian tissue/organs by injection of Ad5 containing the green fluorescent protein (GFP) gene into blood vessels. At 2 d of post-injection, a strong GFP signal was predominantly identified in the heart of chicken embryos injected at Hamilton–Hamburger (HH) 14, 15, 16 and17 stages with the percentages (44%, 53%, 25%, and 14%, respectively) of GFP positive embryos. In quail embryos, the injection at the HH 15 resulted in heart-specific expression of GFP. Western blot analysis revealed that GFP was exclusively expressed in the avian hearts. These results suggest that the GFP gene is specifically delivered to the avian embryonic hearts when Ad5 is injected through the blood vessel at HH 14–17. This adenoviral transduction of genes of interest in avian embryonic hearts can provide new models for understanding functions of genetic factors on embryonic heart development and unravel genetic etiology of congenital heart diseases.

## 1 Introduction

Genetic techniques have been employed to study the function of genes that could provide foundations to ultimately improve productive performance, quality of products, and animal health. The genetic techniques include viral and nonviral transfections of target genes into primordial germ cells *in vitro* and *in vivo* to generate transgenic chickens and quail (Ahn et al., 2015; Chen et al., 2019; Kim et al., 2020). In addition, *ex ovo* electroporation of chicken embryonic tissues and organs has been successfully employed to introduce target genes (Williams et al., 2021; Higgs et al., 2024). The potential impacts of genetic tools have been demonstrated through diverse research in poultry science and developmental biology. Gene transfers *in vitro* and *in vivo* in particular have been demonstrated in poultry and has provided direct function of specific target genes. In recent decades, recombinant adenoviruses have been used for introducing target genes to various cell types *in vitro* and *in vivo*. Among types of adenoviruses, adenovirus type 5 (Ad5) has successfully dominated in human clinical gene therapy applications. In addition, Ad5 could introduce target genes in mammals including rodents, and pigs (Lee et al., 1999; Everts and Curiel, 2004), showing the ability of gene transfer into a broad range of mammalian species *in vitro* and *in vivo*. Beyond mammalian species, the Ad5 could deliver genes of interest in poultry species, including quails, chickens, ducks, and turkeys, to deliver target genes including GFP for expression of the reporter gene (Shin et al., 2009) and CRISPR/Cas9 for generation of genome-edited poultry (Lee et al., 2019; 2022) as shown *in vitro* and *in vivo* experiments (Shin et al., 2009; Lee et al., 2019; 2022).

Although successful gene delivery to mammalian fetuses *in utero* would significantly advance clinical and developmental biology, many difficulties are associated with visible hindrance to precisely target specific fetal tissues or organs. Avian embryos, however, can serve as an ideal model to study the effects of genes on growth and development of embryonic tissues and organs by adenovirus-mediated gene delivery. By carving a small hole in the shell, a technique known as windowing can be used to observe and manipulate an avian embryo while still inside the egg. While Ad5 can be directly injected into various tissues or organs to express target genes at the sites of injection, tail vein injection of rodents resulted in primary expression at the liver, with minor expression in other tissues including the kidney, spleen, and lung (Everts and Curiel, 2004). In this study, similar to tail vein injection in postnatal rodents, Ad5 was injected into embryonic blood vessels in chickens and quail to test its ability to transfer a target gene into various embryonic tissues. Additionally, it was investigated if delivery of target genes into embryonic tissue was influenced by the timing of Ad5 administration into embryonic circulation.

## 2 Materials and methods

### 2.1 IACUC approval and egg source

Experiments using poultry embryos are exempt from requiring University Institutional Animal Care and Use Committee approval, because avian embryos are not considered live animals by the Public Health Service Policy (ILAR News, 1991). All fertile chicken and quail eggs were obtained from The Ohio State University poultry research facility.

### 2.2 Injection of adenoviral vector into chicken and quail embryos

**FIGURE 1:**
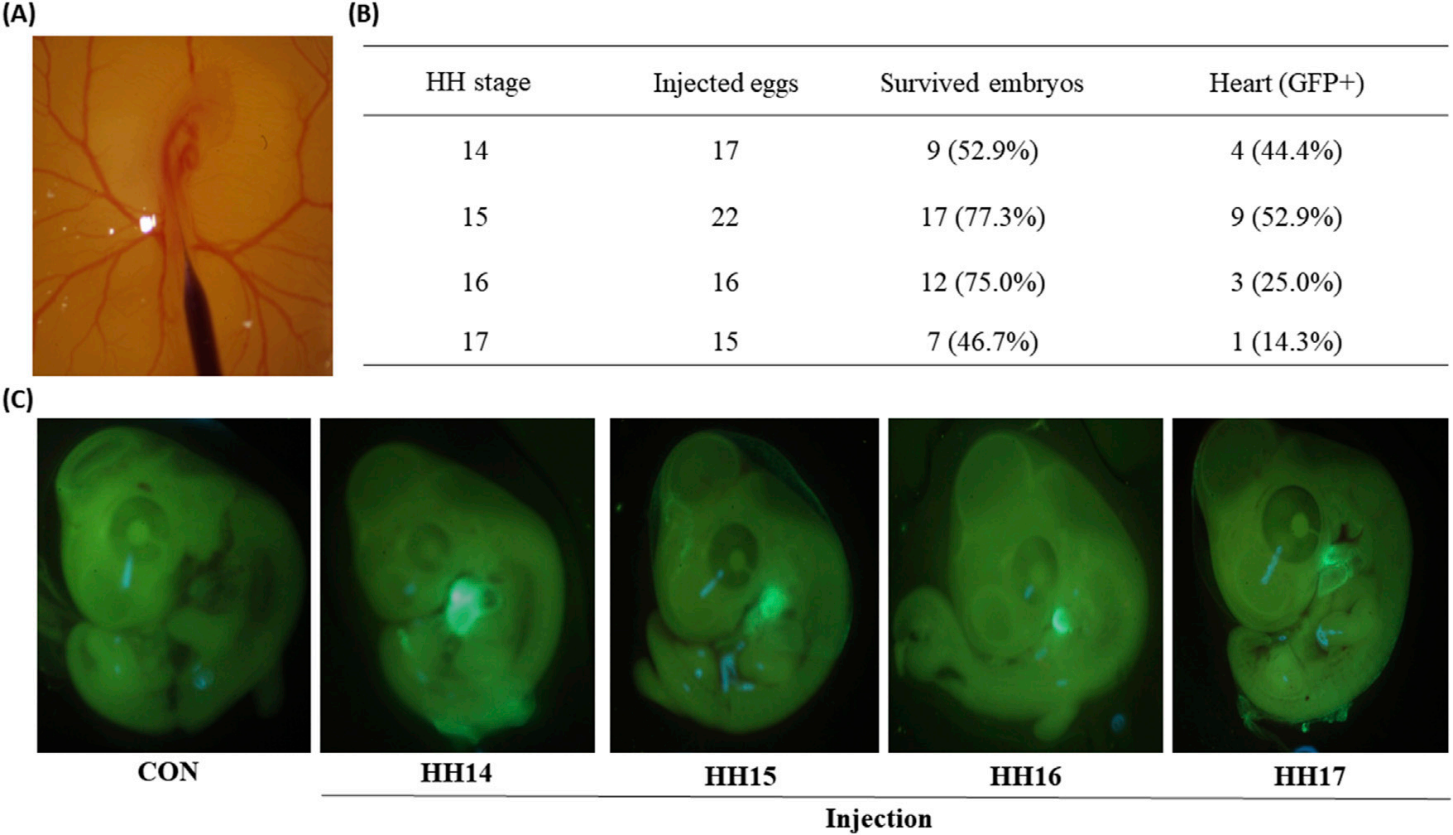
Green fluorescent protein (GFP) expression in chicken embryos of 2 days after the injections at Hamburger–Hamilton (HH) 14–17 stages. **(A)** Microscopic image of injection into the embryonic blood vessel at HH 16 stage. **(B)** Percentages of GFP positive embryonic hearts to the survived embryos injected at different HH stages (14–17). The percentages in the parentheses of the third column were calculated via the following equation [(Survived embryos/Injected eggs) × 100]. The percentages in the parentheses of the fourth column were calculated via the following equation [(Heart (GFP+)/Survived embryos) × 100]. **(C)** Representative fluorescence microscopic images of control embryo and four injected embryos.

An adenoviral vector (Ad5-CBH-GFP) was used for the expression of the green fluorescence protein (GFP) gene under the regulation of cytomegalovirus enhancer and avian β-actin (CBH) promoter. Freshly laid eggs were incubated at a temperature of 37.5°C and 70% relative humidity. After 58, 62, 66, and 72 h (h) [Hamilton–Hamburger (HH) stage 14–17] of incubations of chicken eggs and after 60 h incubation of quail eggs (HH 15), the eggs were taken out from the egg incubator, and eggshells were sanitized with 70% ethanol. The incubation time of 58 h was set as the earliest time to inject Ad5 through a main blood vessel in embryos (Figure 1A). A small window was made on the top of eggs by autoclaved fine forceps (Figure 1A). Microinjection needles were made by pulling microcapillary tubes (504,949, WPI, Sarasota, FL) by a micropipette puller (PC-100, Narishige, Amityville, NY), and grinded by a micropipette grinder (EG-44, Narishige, Amityville, NY). After sterilization of microinjection needles under ultraviolet light, approximately 1 μL of Ad5-CBH-GFP (1.0 × 10^7^ pfu/μL) was injected through a main blood vessel under a SZ61 stereomicroscope (Olympus America Inc., Center Valley, PA) using a microinjection syringe pump (MICRO2T SMARTouch™ controller, WPI, Sarasota, FL) as shown in Figure 1A. After sealing the window with paraffin film, the injected eggs were incubated for an additional 2 d at 37.5°C and 70% relative humidity.

### 2.3 Microscopic observation of GFP in chicken and quail embryos

Two days after administration of the Ad5-CBH-GFP, injected embryos were removed from the eggs and immediately placed on Petri dishes. Embryos were screened for GFP expression using a stereo microscope (SZ61, Olympus) with an external GFP light (DFP flashlights, Nightsea, Hatfield, PA). Images of embryos with GFP expression were acquired using the stereo microscope fitted with a camera (EOS Rebel T7, Canon, Japan).

### 2.4 Western blot analysis

**FIGURE 2:**
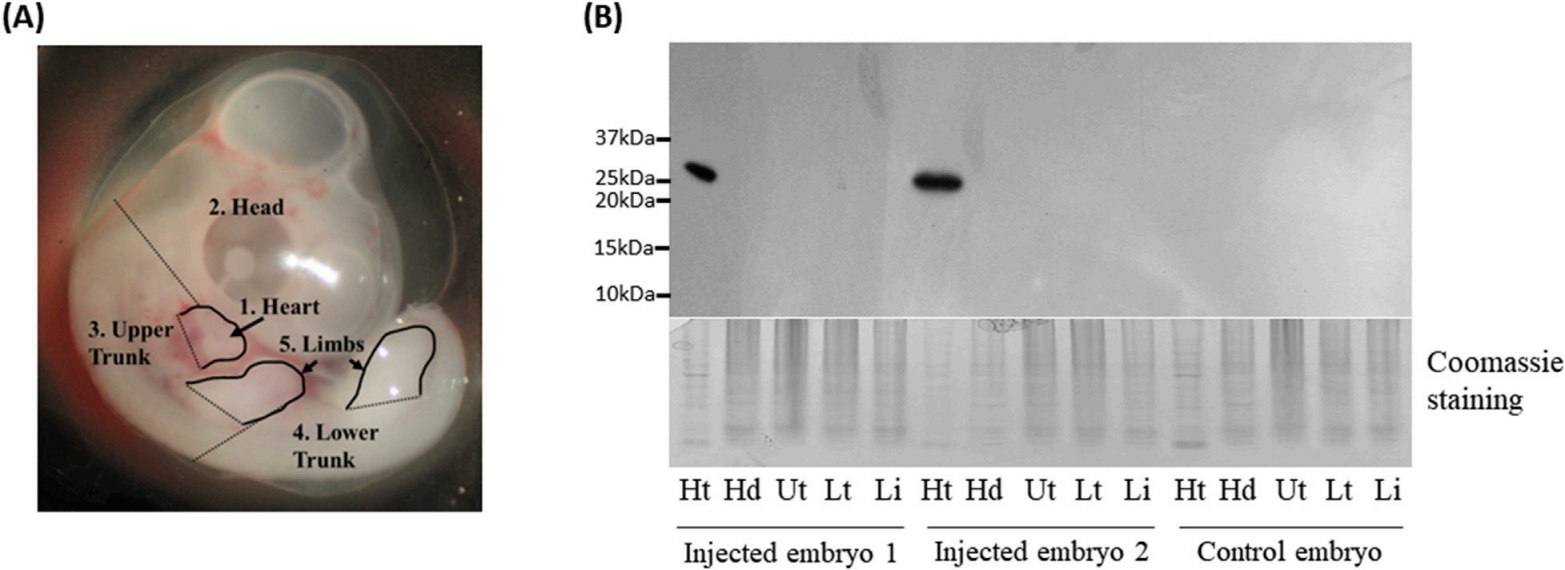
Western blot analysis of Green fluorescent protein (GFP) protein in chicken embryos. **(A)** Five different parts of embryonic tissues dissected for Western blot analysis. **(B)** Western blot analysis of GFP expression in embryonic tissues. Ht: heart, Hd: head, Ut: upper trunk, Lt: lower trunk, Lb: limbs. Coomassie staining was used as a loading control.

Five different tissues, including the heart, head, upper body, lower body, and limb buds (Figure 2A), were collected from chicken and quail embryos 48 h after the injection of Ad5-CBH-GFP into blood vessels. A non-injected chicken and quail embryos of the same age was used as a negative control. All tissue samples were immediately stored at −80°C until further analysis. Detailed procedures for extraction of proteins from tissues and Western blot analysis for GFP protein have been described in our previous report (Woodfint et al., 2017). Extracted proteins were separated in 15% Sodium dodecyl sulfate using a mini-Protein system (Bio-Rad Laboratories, Hercules, CA, USA). Coomassie brilliant blue staining of the gel was used to determine protein loading (Choi et al., 2014). After transferring separated proteins from the gel to the polyvinylidene fluoride membranes, the membranes were blocked in 4% non-fat dry milk dissolved in Tris-buffered saline-Tween for 30 min (min) at room temperature. Then, membranes were incubated for 2 h at room temperature with an eGFP primary antibody (1:3,000 dilution; Clontech, Mountain View, CA, USA). After washing 6 × 10 min in Tris-buffered saline with 0.1% Tween^®^ 20 detergent, the membranes were incubated in horseradish peroxidase-conjugated secondary anti-mouse immunoglobulin G (1:5,000 dilution; Jackson ImmunoResearch Laboratories Inc., West Grove, PA, USA) at room temperature for 1 h. After washing 6 × 10 min in Tris-buffered saline with 0.1% Tween^®^ 20 detergent, specific GFP bands were detected with Amersham ECL plus Western blotting Detection Reagents (GE Healthcare Biosciences, Pittsburgh, PA, USA). The blots were exposed to Hyperfilm (GE Healthcare Biosciences) to visualize the target proteins.

## 3 Results

### 3.1 Transduction of GFP reporter gene in avian embryoinc heart by administration of recombinant adenovirus type-5 vector into embryonic circulation

In this study, the earliest time window for adenoviral injection into embryonic circulation was chosen for the HH 14–17 stages because the dorsal aorta is clearly visible, and embryos are dynamically developing in these stages. Administration of the Ad5-CBH-GFP adenoviral vector into circulation at four different embryonic stages (HH 14, 15, 16, and 17 stages) resulted in percentages of survived embryos, 53%, 77%, 75%, and 47%, respectively (Figure 1B). These survival rates around/over 50% indicate the procedures are not invasive and can yield reasonably high numbers of viable embryos.

**FIGURE 3:**
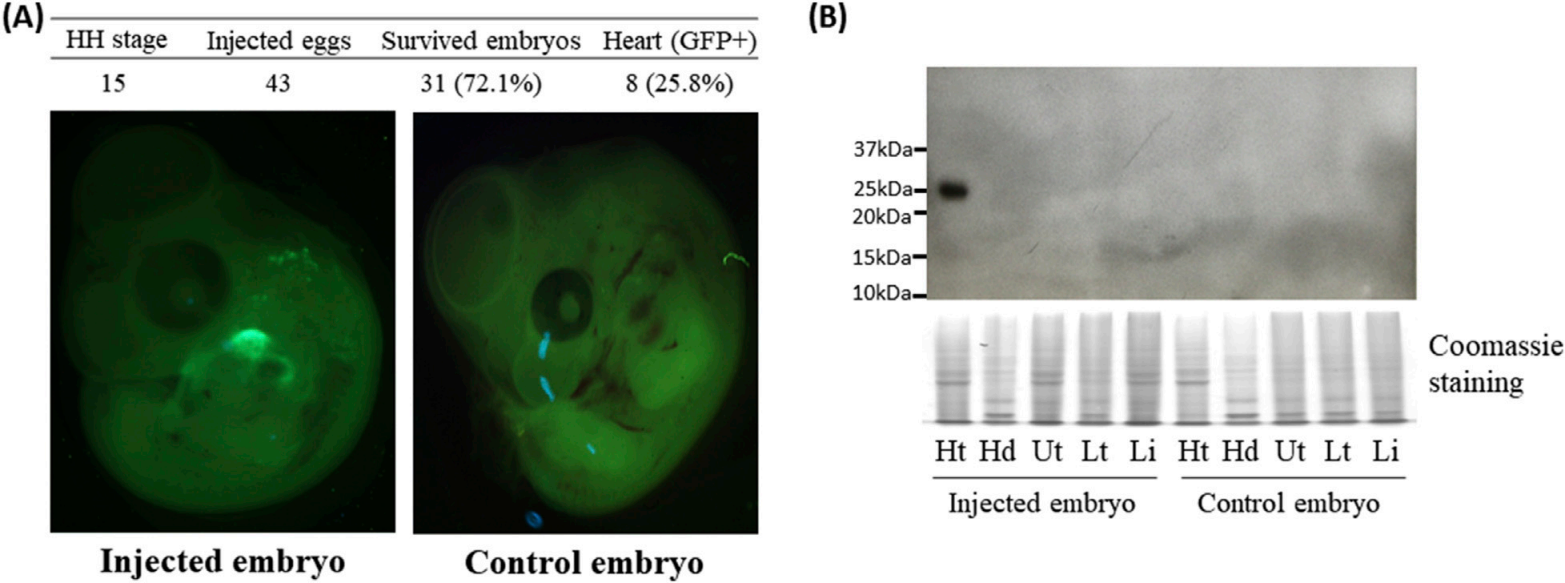
Green fluorescent protein (GFP) expression in the heart of quail embryos 2 days after the injection at the Hamburger–Hamilton (HH) 15 stage. **(A)** Representative fluorescence microscopic images of control and injected embryos. The percentages in the parentheses of the third column were calculated via the following equation [(Survived embryos/Injected eggs) × 100]. The percentages in the parentheses of the fourth column were calculated via the following equation [(Heart (GFP+)/Survived embryos) × 100]. **(B)** Western blot analysis of GFP expression in quail embryonic tissues. Ht: heart, Hd: head, Ut: upper trunk, Lt: lower trunk, Lb: limbs. Coomassie staining was used as a loading control.

Because Ad5 is well known for epichromosomal expression of target genes without integrating viral genome into the host genome (Lee et al., 2019), rapidly dividing cells such as embryonic cells can lose/dilute the adenoviral vectors during cell division of infected cells. In this study, GFP expression in embryos was determined at 2-days post infection. Surprisingly, examination of the survived embryos under the GFP light revealed that strong GFP signals were predominantly found in embryonic hearts (Figure 1C) with the percentages of 44%, 53%, 25%, and 14% from injections at the HH 14, 15, 16, and 17 stages (Figure 1B), respectively. Among these time points, high percentages of GFP expression in the heart of embryos injected with Ad5 at HH 14–15 suggests that there might be a permissive time window for adenoviral transduction. In quail, injection of the Ad5-CBH-GFP adenoviral vector into embryonic blood vessel at the HH 15 stage resulted in survival rate of 72% and a heart-specific GFP expression rate of 26% (Figure 3A).

### 3.2 Heart-specific expression of GFP protein in chicken embryos injected with adenoviral vector containing GFP gene into circulation

To further confirm the GFP expression in the heart, GFP protein was detected by Western blot using GFP antibody. One embryo each for chicken and quail that was not injected served as a negative control. Among the injected embryos, two chicken embryos along with one quail embryo that showed GFP signal in the hearts (Figures 1C, 3A) were selected as representative samples.

The embryos were dissected into the head, heart, upper body, lower body, and limb buds (Figure 2A). In the two injected chicken embryos and one injected quail embryo, protein lysates from hearts showed at the expected size of 25 kDa bands, but not in other tissues, whereas the negative control embryos did not show any GFP protein in all tissues including the heart (Figures 2B, 3B). The results from Western blot analysis further confirmed exclusive expression of GFP protein in the heart.

## 4 Discussion

Because recombinant adenovirus has been designed to be a replication defective and non-integrating DNA viral vector, Ad5 is mostly considered safe and effective for use in gene therapy (Lee et al., 2017). In addition to its clinical application, Ad5 has also been actively used in other mammalian species, including mice, rats, and pigs (Duncan et al., 1978; Lee et al., 1999; Boquet et al., 2008) and avian species for gene editing *in vivo* (Lee et al., 2019; 2022), or overexpression of target genes *in vitro* (Shin et al., 2009). Recently, we demonstrated capability of Ad5 injection into embryonic tissues to express CRISPR/Cas9 in the sites of injection capable of gene editing in those tissues (Lee et al., 2023). In the current study, we first tested whether administration of Ad5 through blood vessels of embryos at HH 14–17 stages would deliver the GFP reporter gene in specific embryonic tissues. GFP images of whole chicken and quail embryos that were injected with Ad5-CBH-GFP adenoviral vector into circulation revealed predominant GFP signals in the heart. In addition, Western blot analysis for GFP protein further confirmed exclusive expression of GFP protein in the heart. Among the time points including HH 14–17 stages, Ad5-CBH-GFP adenoviral vector could more efficiently transduce hearts at the earlier stages (HH 14 and 15), suggesting a time-window for adenoviral transduction in theavian embryonic heart. These data support, for the first time, the predominant targeting of the embryonic heart when an adenoviral vector is administered into the blood vessel of chicken and quail embryos. Although further studies will be needed to explain how the circulating Ad5 predominantly transduces into the embryonic heart, it is possible that embryonic hearts consistently collect and pump blood, injected Ad5 in blood could interact with heart-specific receptors, likely allowing adenoviral transduction of the heart.

Results from the current study showing heart-specific transduction by Ad5-CBH-GFP are somewhat different from what was reported from the mouse study where intravenously administered adenovirus infected mostly the liver (Everts and Curiel, 2004). Although chicken and quail embryos at these early stages do not have a visible liver, it will be interesting to investigate whether administration of Ad5 into circulation of post-hatch chickens and quail would induce expression of a target gene in the liver. Recently, adenoviral vectors containing CRISPR/Cas9 to target *melanophilin* gene have been directly injected into the cervical flexure or limb bud in quail embryos, resulting in change in feather color from brown to gray, a clear phenotype in the injection sites of post-hatch quail (Lee et al., 2023). With this evidence, the Ad5-CBH-GFP can be directly injected into other target tissues to deliver the gene of interests. The current study provides proof-of-concept for capability of the Ad5 in delivering target genes into developing embryonic heart. The Ad5-CBH-GFP especially could efficiently transduce hearts at the earlier stages (HH 14 and 15) and maintain expression of GFP up to the HH 24 stage. These embryonic stages are critical time points for dynamic processes of heart development including looping, cell proliferation and migration, and septation and chamber formation (Wittig and Munsterberg, 2016). Without generating transgenic animal models which require skills, time and resources, these simple tool kits could be used to delivery potential genes including heart-specific novel genes (Ahn et al., 2020) to understand roles of these genes in early heart development. In addition, allowing hatching of the injected embryos will provide avian models for further investigation on the effects of modulations of genes in embryonic hearts on cardiac functions in post-hatch poultry.

Through the current study, we established a genetic tool for heart-specific expression of a target gene in avian embryos. This genetic tool can provide chicken and quail embryonic models with heart-specific expressions of target genes to understand functions of genes in embryonic development of the heart and genetic etiology of congenital heart failure. In the broiler industry, heart failure with sudden death syndrome (SDS) has been known to be caused by genetic mutations in CASQ2 and RYR2 genes (Basaki et al., 2016; 2019). It will be interesting to investigate whether modulation of expression of these genes by Ad5 can affect SDS, eventually leading to confirming or identifying a responsible gene for SDS and using this gene as a selection marker against the population of breeders with SDS.

## Data Availability

The raw data supporting the conclusions of this article will be made available by the authors, without undue reservation.
